# Nanostructured Polypyrrole-Based Ammonia and Volatile Organic Compound Sensors

**DOI:** 10.3390/s17030562

**Published:** 2017-03-10

**Authors:** Milena Šetka, Jana Drbohlavová, Jaromír Hubálek

**Affiliations:** 1 Central European Institute of Technology, Brno University of Technology, Purkyňova 123, 612 00 Brno, Czech Republic; milena.setka@ceitec.vutbr.cz (M.Š.); hubalek@feec.vutbr.cz (J.H.); 2 Faculty of Electrical Engineering and Communication, Brno University of Technology, Technická 10, 616 00 Brno, Czech Republic

**Keywords:** gas sensor, VOC sensor, nanostructures, conductive polymers, polypyrrole, polyaniline, ammonia, ethanol

## Abstract

The aim of this review is to summarize the recent progress in the fabrication of efficient nanostructured polymer-based sensors with special focus on polypyrrole. The correlation between physico-chemical parameters, mainly morphology of various polypyrrole nanostructures, and their sensitivity towards selected gas and volatile organic compounds (VOC) is provided. The different approaches of polypyrrole modification with other functional materials are also discussed. With respect to possible sensors application in medicine, namely in the diagnosis of diseases via the detection of volatile biomarkers from human breath, the sensor interaction with humidity is described as well. The major attention is paid to analytes such as ammonia and various alcohols.

## 1. Introduction

Recently, the gas sensors have found a new potential in medical applications such as detection of various volatile organic compounds (VOCs), which can be considered as biomarkers related to different diseases—especially pulmonary ones [1,2]. Their biggest advantage comes from the non-invasiveness of patient screening via VOC breath-prints produced mainly through changes in specific biochemical pathways in the body [3]. This attractive research direction with huge social impact motivated many researchers to develop novel sensing material which will be suitable for such application, i.e., the detection of extremely low gas concentrations and VOCs in exhaled human breath.

The sensitivity and mainly selectivity of any sensing layer strongly depend on the overall sensor construction and type of a specific material which is used for their fabrication. Nanomaterials have undoubtedly gained the most attention among scientists since they offer high surface to volume ratios resulting in higher sensitivity, versatility of their surface modification towards detection of chosen VOC analytes, and last but not least, relatively easy fabrication methods [4,5]. Among different materials, metal [6] and metal oxides [7] represents the most popular approaches for fabrication of sensing layers in these systems. On the contrary, conducting polymers (CPs) either individually or in a hybrid form, i.e., in the combination with other materials such as various noble metals as silver [8,9], gold [10], palladium [11], metal oxides e.g., ZnO [12], SnO_2_ [13], carbon-based materials such as carbon nanotubes [14,15] or various form of graphene [16,17], are still rarely studied. The functionalization of polymers with the abovementioned materials was found to have a significant effect on enhanced sensor performance, especially via improved adsorption of organic compounds and via detection of low concentration VOCs. Therefore, CPs represent an important class of functional organic materials for next-generation electronic and optical sensors [18,19]. However, the polymers are unstable at the nanometer scale due to the nature of covalent bonds which makes their nanostructures unstable as well. Because of this, so far progress in the synthesis of polymer nanomaterials has been relatively slow, and only limited research has been conducted on the fascinating properties polymer nanomaterials possess, in contrast to inorganic nanomaterials.

Comparing the number of scientific works devoted to ammonia and VOC sensors, there is a significant lack of information about the sensor concept based on polymer sensing materials which needs to be covered. Therefore, we provide this concise and didactic review on the recent development of different polymer-based sensors with a main focus on polypyrrole (PPy). Following a general introduction, we summarize the latest attempts of researchers focusing on the fabrication of such sensors according to the polymer used for the sensing layer in the first section and then according to target analytes in the second section. We also show an example of sensor interaction with ammonia and VOC analytes via an appropriate “sensing” model and we describe their interference with humidity as well, which represents one of the major challenges in the practical application in detection of VOCs in breath. To conclude, we provide a discussion about sensor limitations as well as biggest achievements so far and showed the promising future perspectives.

## 2. Basic Characteristics of CP

**Figure 1:**
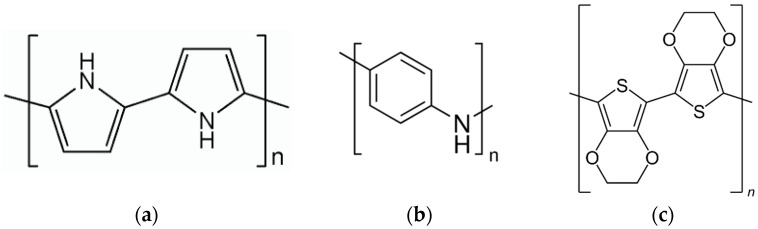
Structures of some conductive polymers: (**a**) PPy; (**b**) PANI and (**c**) PEDOT.

From the material point of view, CPs such as PPy [20,21], polyaniline (PANI) [22,23], polydiacetylene (PDA) [24,25] and various derivates of polythiophene like poly(3,4-ethylene-dioxythiphene (PEDOT) [26,27] are the most promising materials for gas sensing applications. The structures of PPy, PANI and PEDOT are illustrated in Figure 1. It can be seen that all of them have a heteroatom in their monomer units, namely sulphur in the case of PEDOT and nitrogen in the case of PPy and PANI. The list of suitable polymers also includes some less common compounds, for example poly(vinylidene fluoride-hexafluoropropylene) [15] and polydiallyldimethylammonium chloride [28,29].

In general, the main advantage of all the abovementioned polymers is their capability of working at room temperature, which make them outstanding compared with other materials, for instance metal and metal-oxides which require very high temperatures (from 200 °C [30,31,32] up to 400 °C [33,34], or even higher [35]). Moreover, CPs have been in the spotlight of research investigations, especially in the field of chemical sensors, thanks to their high conductivity which results from π-electron conjugation [36,37]. The aromatic ring of CPs consist of chains with single and double bonds, which lead to broad π-electron conjugation. Basically, the sensitive and rapid response to various chemical species is caused by the oxidation level of the CP, which is readily affected by chemical or electrochemical doping/de-doping (oxidation/reduction) mechanisms between the CP and the exposed analytes [38]. Other benefits which make CP significant in the range of sensing materials are:

- the fast charge-discharge mechanism which is related directly with polymer structure, i.e., the presence of conjugated bonds, and applied voltage,
- high charge density causing high conductivity,
- solid stability in ambient conditions,
- the physico-chemical properties of the systems which are not easily changed by an external stimulus,
- and last but not least easy a low cost way of preparation [39].

CPs with nanometric features have demonstrated improved properties, similarly to other nanoscaled materials like metal or metal oxides [40]. For instance nanoscaled CPs are more conductive and sensitive compared with bulk materials owing to their higher surface area and conductive pathways, what is reflected in a rapid adsorption and absorption/desorption kinetics for analytes [41,42,43]. Due to this high electrical conductivity and fast electron transport, CPs have also received great attention in the field of electrochemical high power energy storage devices such as supercapacitors [44]. Here, CPs are often combined with other materials, namely metal [45], metal oxides [46,47,48], and carbon materials such as carbon nanotubes and graphene [49,50,51]. 

According to the literature survey, the most common characterization of CP structures is performed using Fourier Transformed Infrared (FT-IR) spectroscopic analysis [21,52,53]. Since the review is focused on PPy, we summarize the observed FT-IR peaks for PPy characteristic vibrations from different published works in the Table 1. The corresponding peaks for PPy appear in the range of 700–3000 cm^−1^. 

## 3. Preparation of PPy Sensing Layers

PPy is a p-type organic conductive polymer created by oxidation of pyrrole monomer, further denoted as Py (Figure 1). The synthesis of PPy is relatively easy due to the oxidation of Py, its water solubility, great adherence to different types of substrates, and commercial availability. Nanostructured PPy materials have been synthesized in the form of nanoparticles [58,59], nanowires [21,60], nanorods [10], nanosheets [61], nanotubes [53] and nanoribbons [62]. In general, the methods for nanostructured polymer preparation can be classified according to type of reaction: electrochemical (see Section 3.1) or chemical (see Section 3.2). Few examples of these methods are listed in the Table 2. 

In both types of synthetic approaches the polymer nanostructures can be prepared using template and template-free methods. The researchers usually distinguish among three different template methods, namely hard-template synthesis, soft-template synthesis [53,63,64], and reactive template synthesis [65]. One of the most used hard templates is nanoporous anodic alumina oxide (AAO) which provided uniform dimension of pores, high density, high aspect ratio and controllable diameter of nanostructures. AAO template can be used either for chemical [60] or electrochemical synthesis [39] of PPy nanostructures (such as nanowires, nanorods or nanotubes). In addition, it can be also used for the synthesis of organic–inorganic hybrid materials, where the PPy nanostructures are combined with an inorganic materials, such as Au-PPy nanorods [10] or CdS-Ppy nanowires [66,67].

Nowadays, the research trends in the field of sensors, including gas and VOC sensors, are focused on immobilization of sensing layers on various flexible substrates like papers or plastics. Beside traditional cellulose paper, the researchers also tested vellum paper, filter paper and various photopapers as potential substrates for flexible gas sensors. These attractive flexible sensors represent a new alternative technology for fabricating simple, low-cost, portable and disposable analytical devices [72,73]. However, the number of works about CPs deposited on these types of substrate and their application in the gas-phase sensing is still limited. Most flexible gas sensors are based on PANI [74,75,76]. Less attention is paid to PPy [71], eventually poly(*m*-aminobenzenesulfonic acid) [77], poly-diallyldimethylammonium chloride (PDDAC) [28,29] or polydiacetylene (PDA) [24]. 

**Figure 2:**
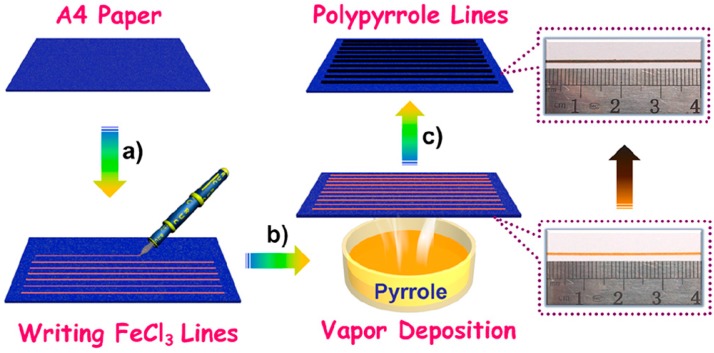
Schematic illustration of pen-writing PPy on A4 paper: (**a**) pen-writing FeCl_3_ solution on the paper; (**b**) Exposing the FeCl_3_ lines to Py vapor; (**c**) Interfacial polymerization of Py along the FeCl_3_ lines. Insets are photographs of A4 paper written with FeCl_3_ and PPy after fumigation. Reprinted with permission from [71]. Copyright (2017) American Chemical Society.

An interesting example of flexible gas sensor with PPy was developed by Jia et al. [71]. They used a simple and low-cost “pen-writing” method for immobilization of PPy on cellulosic paper (Figure 2). The pen-written paper chip with excellent mechanical and electrical properties was then used for in situ detections for ammonia with limit of detection (LOD) as low as 1.2 ppm.

### 3.1. Electrochemical Synthesis

In this method, the synthesis of PPy is based on the polymerization of the Py on electrodes by electrochemical oxidation. From the group of electrochemical synthesis of PPy, one of the well-known synthesis is the electropolymerization, which can be performed using different modes such as galvanostatic (under constant current), potentiostatic (constant potential) [21], potential sweeping techniques such as cyclic voltammetry or other potentiodynamic methods [78].

The final properties of the structures synthetized via electropolymerization of Py are strongly dependent on the Py concentration [79], type of solvent [80,81], applied voltage and current density [82], pH of electrolyte [83] and its temperature [84,85]. The thickness of the deposited material depends on the integrated charges used for electrochemical synthesis [39]. The deposition of PPy is achieved on the conductive surfaces that serve as working electrodes, for this purpose, thin conductive metallic layer such as gold, which can be sputtered or vapour deposited on one side of the membrane, are typically used [86]. The electrochemical cell consists of three electrodes: a conductive metal layer acts as a working one, a platinum sheet as counter electrode, and reference electrode is usually made of Ag/AgCl. The reaction is carried out in an electrolyte solution, which is a mixture of Py monomer, dissolved in an appropriate solvent containing the desired anionic doping salt [87]. After applying a certain voltage in potentiostatic mode or constant current, the polymerization of Py is started. The set values of both magnitudes depend undoubtedly on the size of treated sample, distance between electrodes and their area. 

According to Sadki et al., PPy can react by different mechanisms during its electro-polymerization, namely the Diaz, Kim, Pletcher, and Reynolds ones [88]. The Diaz mechanism, which is based on the creation of PPy via coupling of two resonant forms with unpaired electrons, is the most frequently used in the literature [89] and therefore we will describe it in more details. The readers may find detailed information about the other mechanisms in the above mentioned review [88]. 

**Figure 3:**
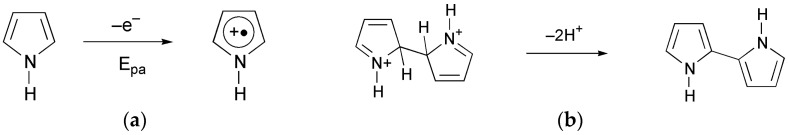
The schematic illustration of Py dimer’s synthesis: (**a**) formation of the cation radical R^+•^ and (**b**) formation of the aromatic dimer. Reproduced from [88] with permission of The Royal Society of Chemistry.

**Figure 4:**
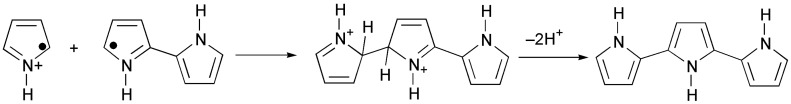
The schematic illustration of Py trimers’s synthesis. Reproduced from [88] with permission of The Royal Society of Chemistry.

During the first step of Diaz mechanism the oxidation of monomer and its transformation into the cation radical R^+•^ proceed at the applied voltage, see Figure 3a. The radical appeared in the several resonance forms. The reaction between two radicals with unpaired electrons results in formation of bonds between them and creation of the dihydromer. The loss of two protons of dihydromer results in creation of the aromatic dimer, the reaction is illustrated in Figure 3b. The next step is the oxidation of dimer and creation of the dimer radical. This oxidation is happening faster than oxidation of the monomer, because the unpaired electron is now delocalized over the two rings, and needs lower oxidation potential for creation of radicals. The appropriate resonance form of dimer reacts with suitable resonance form of monomer and they are giving the structure, which after the loss of two protons, makes the neutral trimer, the reaction is presented in Figure 4. The described steps are repeating in the same order (creation of radical, coupling, and deprotonation) until the final polymer structure is obtained. The final PPy structure is oxidized conducting form with a positive charge after every three to four Py units. This charge is then counter-balanced by an electrolyte anion. The Diaz mechanism confirmed the main fact about PPy, existence of a p-type radical [88].

**Figure 5:**
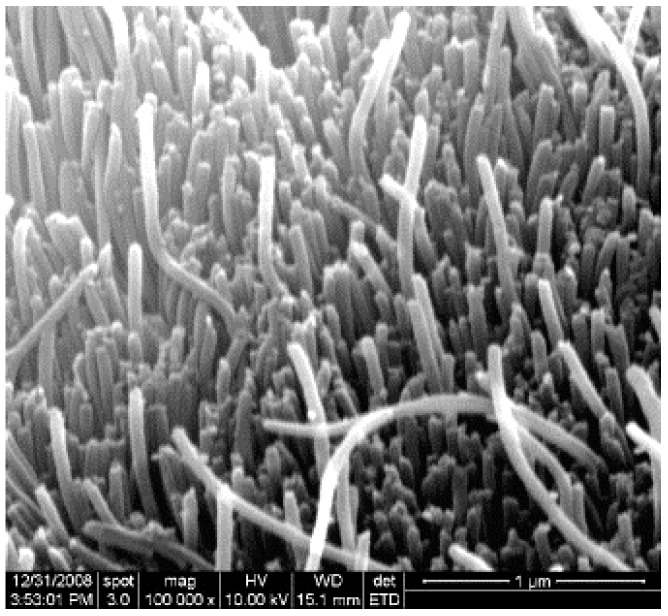
SEM image of the PPy nanowires grown in the AAO template. Reproduced from [21]. Copyright (2017), with permission from Elsevier.

Concerning template-assisted electrochemical synthesis, Zhang et al. obtained PPy nanowires with diameters about 50 nm using the AAO template via electrochemical polymerization of Py (Figure 5) [21]. The three electrode system was used for the synthesis of the PPy nanowires. The electropolymerization process of Py was carried out in the solution of Py with lithium perchlorate (LiClO_4_), which served as the oxidizing agent. 

**Figure 6:**
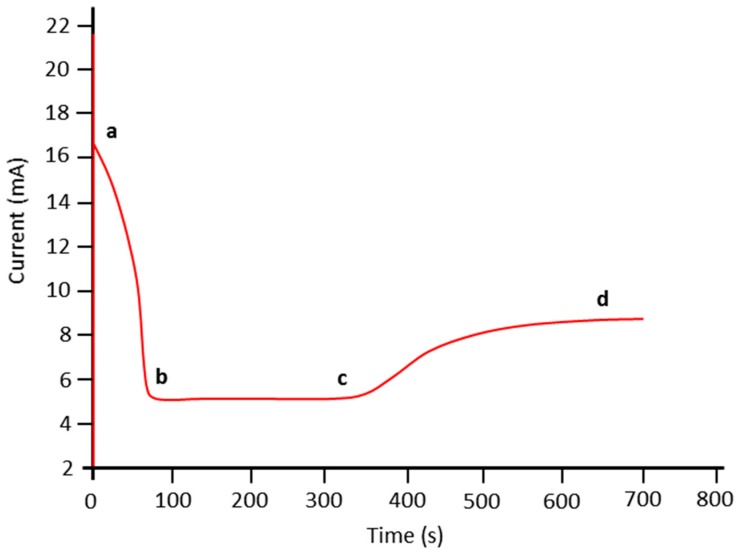
Current-time curve during PPy synthesis. Reproduced from [21]. Copyright (2017), with permission from Elsevier.

The applied voltage during the electropolymerization was 1 V. The reaction time was 650 s. The final structure consisted of the PPy nanowires with the thin PPy layer formed on the top of the nanowires. The authors explained the changes in the current at the constant potential during the growing of the PPy nanostructures passed through three different stages: (1) creation of the PPy nucleus; (2) PPy nanowires; and (3) thin PPy film. The beginning of the electropolymerization is followed by the decreasing of the current (see Figure 6, from point a to b) which corresponds to formation of PPy nucleates. After the first step is finished, the current decreased to certain point and stayed constant (from b to c) till the all AAO pores are filled with the PPy, more precisely till the moment when the nanowires are created. In the last stage the current started to increase again (from point c to d), manifesting the growth of the PPy out of the AAO pores and finally the creation of the thin PPy film on the top. Subsequently, the created structures were sputter-deposited with gold layer from both sides, which had the function of the electrodes and the created chemiresistive sensor was used for the detection of ammonia. 

**Figure 7:**
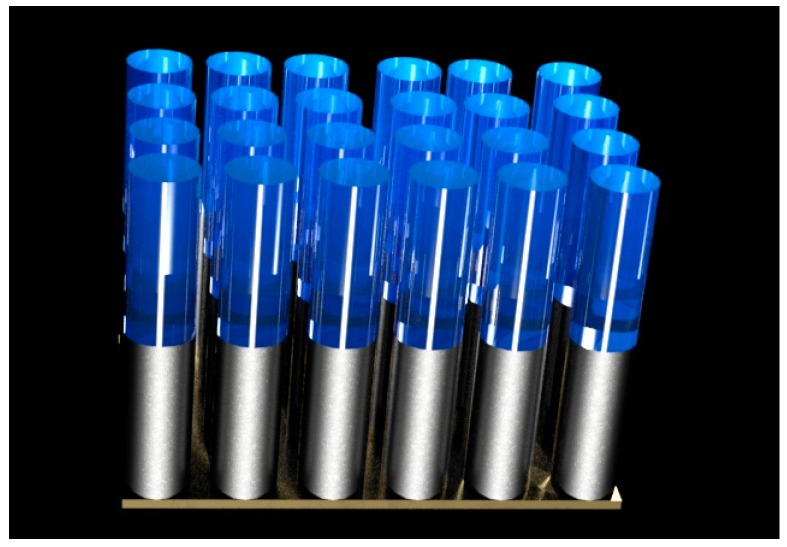
Schematic illustration of Au-PPy nanorods.

Lee et al. obtained the metal-organic framework, which is based on gold-polypyrrole (Au-PPy) nanorods with a diameter of about 200 nm [10]. In the first step, they synthesised gold nanorods via electrodeposition into AAO nanoporous template and in the second step, they deposited the PPy nanorods via electropolymerization of the Py. The electropolymerization reaction was carried out in Py solution dissolved in acetonitrile, where tetraethylammonium tetrafluoroborate was used as the oxidizing agent. The applied voltage was 0.95 V. The Au-PPy nanorods (see Figure 7) served as the sensor for the detection of the VOC (namely of acetic acid, benzene, and toluene).

### 3.2. Chemical Synthesis

Compared with the electrochemical synthesis of the PPy, the chemical method is simpler because it does not require any special instruments [36,90,91]. The type of surfactant, e.g., sodium dodecylbenzene sulfonate [92], and the oxidizing agent are the most responsible for the polymerization of Py and the final conductivity of PPy. The researchers made attempts to increase the electrical conductivity of PPy by using various dopants, namely lithium perchlorate (LiClO_4_), *p*− toluene sulfonate and naphthalene sulfonic acid [69].

Common oxidants used for Py polymerisation include ferric chloride, ferric perchlorate and ammonium peroxydisulphate. According to the literature, ferric chloride (FeCl_3_) is the most used oxidizing agent [93,94,95]. The reaction path is simple: after the addition of the Py in the aqueous solution of FeCl_3_, the anions from the solution come into contact with the neutral polymer matrix changing it into Py^+^ cations spontaneously. These Py cations are then participating in the polymerization and formation of black PPy [96]. 

**Figure 8:**
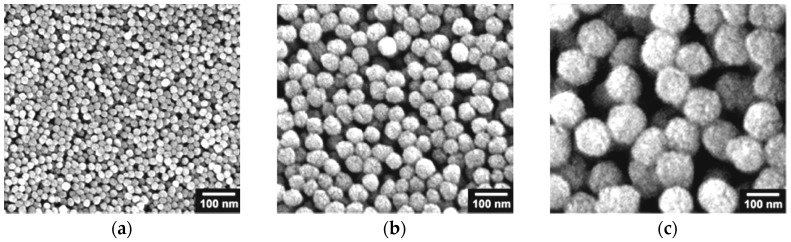
SEM images of PPy nanoparticles with diameter of (**a**) 20 nm; (**b**) 60 nm; and (**c**) 100 nm. Reprinted with permission from [58]. Copyright (2017) American Chemical Society.

Kwon et al. synthesized the PPy nanoparticles with different diameter size (20, 60, and 100 nm) by the polymerisation of the Py via its chemical oxidation [58,59]. The synthesis is based on the reaction between water-soluble polymers (polyvinyl alcohol dissolved in the water) and metal cations (FeCl_3_) in aqueous solution. These compounds form the complex, which has the main role in the oxidizing process of the Py monomer. In particular, FeCl_3_ behaves as the oxidizing agent which initiate the chemical oxidation of Py, while polyvinyl alcohol (PVA) acts as a self-assembled precursor. PVA is responsible for a steric stability during the growth of polymer nanoparticles. The size of nanoparticles is related with appropriate concentration of these two reactants which are mixed and dissolved in water. After addition of the Py to this mixture and its contact with FeCl_3_, the oxidation of Py and its polymerization reaction begin. This is represented by the creation of black solution which corresponds to created PPy nanoparticles (Figure 8). Therefore, the advantage of this synthesis of PPy compared with others methods which will be described in next section, lies in no extra requirements for special surfactants or specific templates.

Less common chemical synthesis approach of PPy is based on vapour phase polymerisation [70]. The method involves application of the oxidant to the surface using a solvent coating process and subsequently exposure of the coated surface to the vapour of the monomer. This vapour phase polymerization was initially described by Mohammadi et al. where the authors used FeCl_3_ or H_2_O_2_ as oxidants in order to form PPy films [97]. 

Hernandez et al. used an AAO template for chemical polymerization of PPy nanowires [60]. The authors used a previously prepared AAO template, which they immersed in the cold Py solution and they added FeCl_3_ as the oxidizing agent for chemical polymerization of Py. The nanowires were observed after the dissolution of AAO in hydrofluoric acid. The dried PPy nanowires were later dissolved in ethanol and dispersed across the microfabricated gold electrodes on SiO_2_/Si substrate. The authors concluded that the nanowires synthesized via chemical polymerization are more ordered compared to electrochemically synthesized ones. 

**Figure 9:**
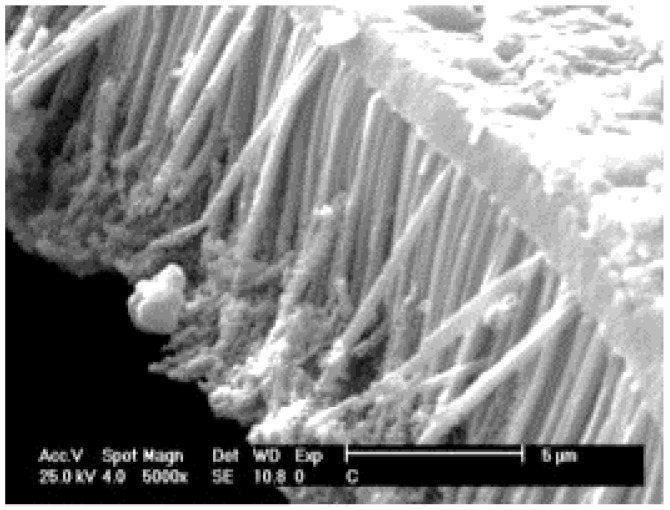
SEM image of PPy nanostructured array prepared by chemical synthesis in AAO template after its dissolving. Reprinted from [98]. Copyright (2017), with permission from Elsevier.

Similar work employing combination of chemical synthesis and template was used by Hassanzadeh et al. for the creation of a PPy nanostructured hydrogen sensor [98]. This gas sensor based on PPy nanowire arrays was fabricated through chemical polymerization in an AAO membrane (Figure 9). The authors used commercial Whatman anodiscs with pore diameter of 200 nm and thickness of 60 µm. They optimized the polymerization time up to 3 h and molar ratio of oxidant (0.2 M FeCl_3_) to Py monomer (0.2 M aqueous solution). This molar ratio provided the highest specific surface area of about 173 m^2^/g. After dissolving the AAO template 10% HF, the obtained PPy nanostructured arrays supported with PPy layers were rinsed and copper wire electrodes connecting the sensing layer with measuring circuit were attached to the opposite ends of PPy sensing array with silver paste.

**Figure 10:**
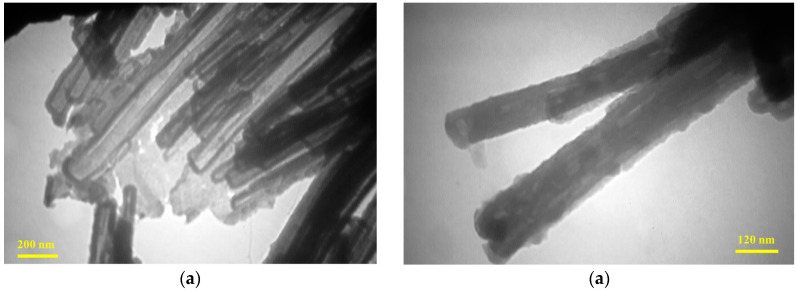
TEM images (**a**,**b**) of synthesized PPy nanotubes with two different magnitudes. Reprinted from [13]. Copyright (2017), with permission from Elsevier.

Yang et al. used the complex of anhydrous FeCl_3_ and methyl orange as a soft template for chemical synthesis of PPy nanostructures [63]. In this approach, a certain amount of the Py monomer was added on the already prepared soft template, and it was stirred for 24 h. FeCl_3_ was responsible for the oxidation in the polymerization reaction of Py and resulted in formation of PPy nanostructures. After finishing the process, the sample was washed and dried. PPy dispersion in water was then drop-casted onto the glass substrate. The same method was used by Joulazadeh et al., where they observed PPy with nanotubular morphology [13,53] (Figure 10). 

**Figure 11:**
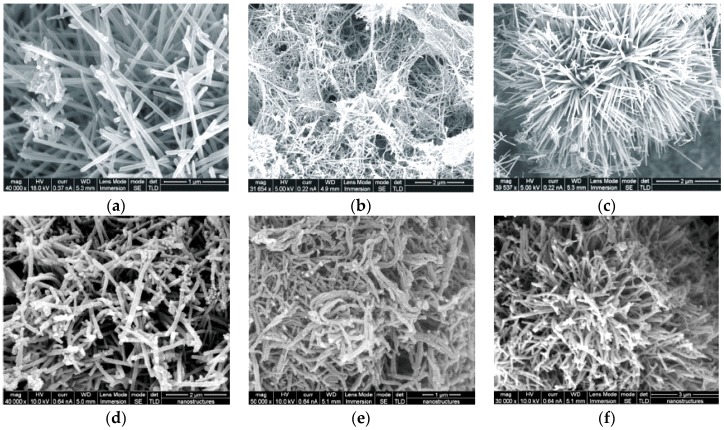
SEM images of three different MnO_2_ nanostructures: (**a**) nanorods; (**b**) nanowires; and (**c**) urchins with corresponding morphologies of PPy: (**d**) nanotubes; (**e**) nanofibers; and (**f**) urchins. Reprinted from [65]. Copyright (2017), with permission from Elsevier.

Dubal et al. used MnO_2_ reactive template with three different morphologies, namely nanorods, nanowires and urchins, for chemical synthesis of PPy nanotubes, nanofibers and urchins [65] (Figure 11). They dispersed MnO_2_ nanostructures in deionized water and then added 1 M HCl K_2_Cr_2_O_7_ and Py monomer. The mixture was stirred and then sonicated. Later, the solution was stirred for 5 h and maintained at room temperature for 24 h. Once the PPy nanostructures were obtained, the MnO_2_ template was dissolved. 

## 4. Gas and VOC Sensing

When talking about the main functional characteristics of sensors, one has to take into account several parameters such as sensitivity, selectivity, stability, response time and recovery time [99]. Depending on the type of transducing mechanism, these parameters may vary a lot. In general, it is expected that the sensitivity of any type of gas sensors must lay in the range of ppm or even ppb. The response time is defined as the necessary time to reach 90% of the total measured electrical magnitude change, and it is as fast, as in units of seconds. The recovery time, which is defined as the time required for the measured electrical magnitude to return to the original value, upon switching from sensor’s exposure of the target gas to 100% carrier gas (syntactic air or some halogen gas) is also expressed in units of seconds. The other important parameter is sensor saturation, which means that dependence of the sensor response on the concentration of analyte is no longer linear [11,53]. In other words, the signal that needs to be measured is larger than the dynamic range of the sensor.

Most of sensors based on CP polymers operate via a chemiresistive transducing mechanism. In comparison with chemiresistive PPy-based sensors, less attention is paid to optical sensors, even though they usually provide excellent sensitivity. Also, some works are related to the PPy sensors which work in an impedance and capacitance mode. Hence, we will briefly describe the working principle of mentioned transducing mechanisms in the following paragraphs: 

*Chemiresistive sensors* belonging to the electrochemical sensors group are based on the measurement of a change in resistance R. It means the sensor response *S* in % is correlated with the change in the electrical resistance before and after interaction with analyte (gas or VOC) using the following equation:

(1) $$S=\frac{R_{gas}-R_{0}}{R_{0}}\cdot 100\%,$$

where *R*_gas_ is the value of the resistance after exposing to the gas, and *R*_0_ after exposing to the carrier gas, respectively. In general, the resistance of sensor is expected to revert to its original value during all response/recovery cycles, indicating that the sensing process is reversible for the sensor.

*Optical sensors* usually measure the changes of optical absorption at specific wavelengths [100]. The sensor response (*ΔA*) can be then calculated as:

(2) $$\Delta A=\frac{A_{gas}-A_{air}}{A_{air}}\cdot 100\%,$$

where *A*_gas_ is value of the absorbance when the sample is exposed to the target gas, and *A*_air_ is value of the absorbance when the sample is exposed to air.

*Capacitance sensors* measure the change in dielectric constant of films between the electrodes as a function of the gas concentration.

**Figure 12:**
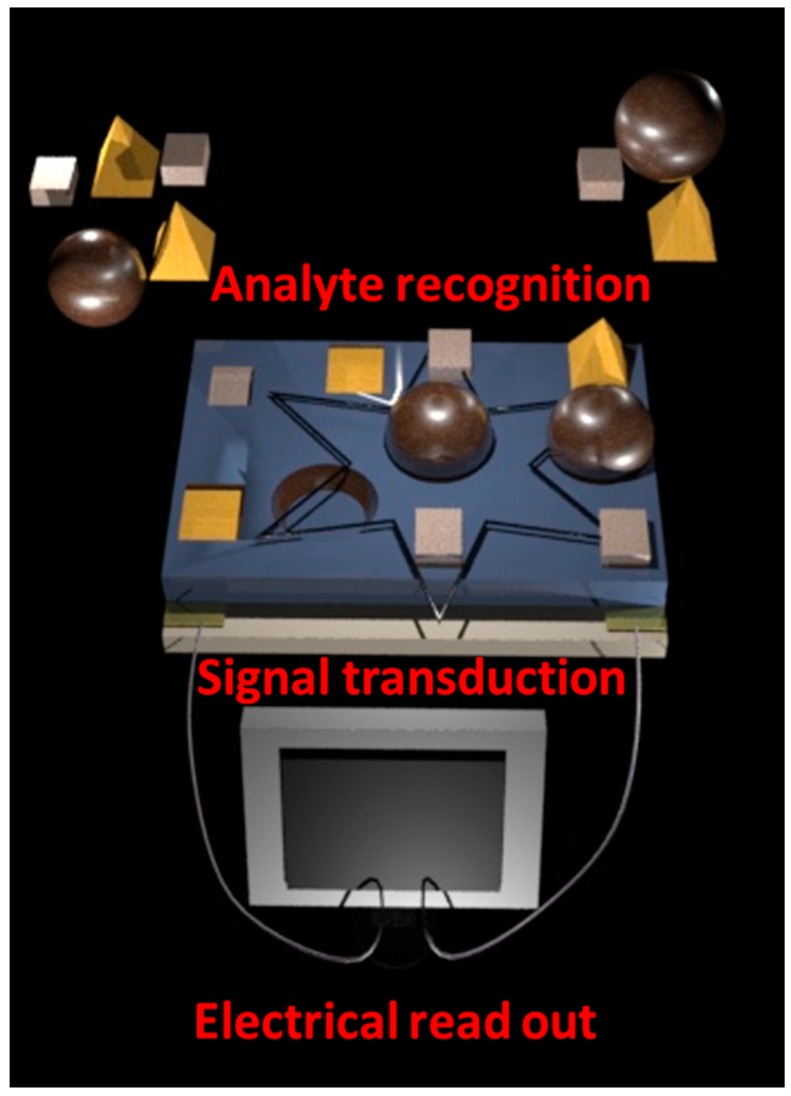
Schematic illustration of an electrochemical sensor consisting of substrate, electrodes, and CP film which is acting as sensing material and transducer. The overall sensing process involves analyte recognition, signal transduction, and electrical readout.

*Impedance sensors* measure the resulting current when a sinusoidal voltage is applied. Impedance as a complex number is then calculated as the ratio of voltage to current in the frequency domain [101]. Impedance sensors read resistive and capacitive part as a dependence on the presence of gas at variable frequency. The technique offers interesting possible benefits in the discrimination of gas and VOC analytes [102,103]. The sensing process of sensors based on CP can be divided into three stages (Figure 12):

1. Recognition of the analytes: the CP nanostructures act as sensitive layer and interact with the analytes with different level of selectivity.
2. Signal transduction: if the sensitive layer recognizes the analytes, it is reflected as a change of electronic charge-transfer properties of the CP. That changes are in the quantitative correlation with the concentration of the analytes [104,105]. The oxidation or reduction reactions proceeding between the sensitive layer and exposed analytes cause a physical swelling of the polymer structure.
3. Electrical readout: finally, the previously described steps are monitored as changes of the electrical resistance or more general of any electrical magnitude.

In the following subsections, we will provide more detailed description of most frequently used types of CP-based gas sensors. Our focus will be on the sensing properties of previously mentioned PPy nanostructures, where we will compare the minimum LOD, sensor response, response time and recovery time, and also perceive the sensitivity to target gases and VOC, especially ammonia, various alcohols and water vapor. In addition, comparison between the sensing performances of the PPy gas sensors and the sensors based on other material, namely metal, metal oxide and carbon base materials, for the detection of ammonia and some most frequently detected alcohols, will be summarized in following part. It will provide the better understanding of the advantages and disadvantages of PPy based sensors.

### 4.1. Detection of Ammonia

PPy behaves like p-type semiconductors via appropriate doping [106] and can affect the change of conductivity through interaction with gas or vapour [107,108]. The sensing mechanism of PPy nanostructures is different for various analytes depending on the presence of their functional groups. According to our rigorous literature survey, we found the highest response of PPy based sensor was recognized for ammonia. Regarding suggested medical application, ammonia can be found in elevated concentrations between 0.8 to 14 ppm in exhaled breath of patients with diagnosis of renal disorders or ulcers, while in normal subjects it is in the range of 0.15–1.8 ppm [62,109]. 

NH_3_ is an electron-donating molecule reducing the holes density in PPy and thus resulting in the increase of the electrical resistance. In opposite, the analytes such as alcohols behave as electron-acceptors because they create new holes in the PPy structure, leading to the decrease in the electrical resistance [41]. In other words, during ammonia sensing the donation of a lone pair of electrons of nitrogen to the initially oxidized PPy results in the decrease in the conductivity and the formation of neutralized PPy [110]. 

The first possible reversible interaction mechanism between the PPy and ammonia electron donating molecules which decrease the doping level of PPy by compensating the effect of the original dopant (denoted here as A^–^), can be described as follows [21,111,112]:

PPy^+^/A^−^ + NH_3_ ⇆ PPy^0^/NH_3_^+^, A^−^, (3)

**Figure 13:**
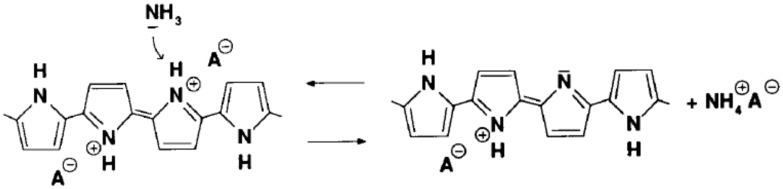
The schematic illustration of PPy interaction with ammonia. Reprinted from [111] with permission from Elsevier. Copyright 2017.

An alternative mechanism for the compensation reaction (see Figure 13), which involves the proton transfer between the polymer and ammonia which is a strong base and can easily attack protons of PPy, could be written as:

PPy^+^/A^−^ + NH_3_ ⇆ PPy^+^(−H)^0^ + NH_4_^+^A^−^. (4)

Similarly to PPy, PANI also interacts reversibly with ammonia which is related to their low redox potentials (−0.2 V and 0.2 V versus SSCE, respectively). An overview of PPy-based sensors, including their response time, recovery time and LOD towards ammonia is provided in Table 3.

The PPy nanoparticles prepared by Kwon et al. [58] were deposited on the gold interdigitated microelectrodes by a spin-coating and a drop-casting method, and used to develop a chemiresistive sensor for the detection of ammonia. The PPy nanoparticles on the top of electrodes were deposited by a spin-coating method which provided a more homogeneous distribution and also higher sensitivity to gases compared to the drop-casting method where nanoparticles were randomly distributed. Significant results were noticed for ammonia, where the sensor showed a LOD close to 5 ppm. The response time of the sensor was less than 1 s. In the case of the recovery time, 30 s was necessary for the sensor to recover after exposure to 5 ppm of NH_3_. 

The same authors described the work of a chemiresistive sensor with a special nanostructure design based on multidimensional PPy nanotubes decorated with nanowires and nanonodules via vapour deposition polymerization [113]. Compared with the nanoparticles which they already described in the previously mentioned paper [58], those specific nanostructures showed an impressive response to NH_3_: the minimum LOD was 10 ppb. 

An ammonia sensor based on PPy nanowires with diameters around 50 nm designed by Zhang et al. [21] was able to respond in the percentage range from 10% to 26% for ammonia concentrations from 1.5 ppm to 77 ppm, respectively. 60 s was enough for the sensor to respond to 77 ppm of ammonia. Comparing the response and response time of the single PPy nanowire sensor from [60], where the response was only about 7% for the concentration of 73 ppm of ammonia and the response time was 10 min, we can notice a marked improvement in the sensor characteristics. 

Beside a polymer nanostructured array, researchers have also attempted to use a single polymer nanostructure as published by Hernandez et al. [60]. A single PPy nanowire placed on microfabricated gold electrodes on a SiO_2_/Si substrate was used for the detection of ammonia. The sensor showed sensitivity to ammonia at concentrations as low as approximately 40 ppm. The authors also monitored the sensor response to NO_2_, but the sensor did not show any sensitivity to this analyte. These results are opposite to those of Chougule et al. who confirmed the sensor response of 14% to 100 ppm of NO_2_ using a PPy film with a granular structure [118]. 

Xue et al. fabricated a single crystal high-oriented PPy nanotube array using an AAO template under low temperature [114]. The growth of the PPy nanotubes on a cold surface allows nice ordered structures. The diameter of the resulting nanowires is about 100–110 nm, where the external diameter of the nanotubes is around 100 nm and the wall thickness about 10 nm. The ammonia sensing properties of this PPy nanotube array have been studied at room temperature. This sensor easily detects ammonia down to 1 ppm, and exhibits very rapid response and recovery times (16 s). The LOD can even reach 0.05 ppb as the highest sensitivity. Therefore, the authors concluded that those ultrahigh performances of PPy nanotubes are the result of not only the hollow structure and high surface area of nanotubes, but also a consequence of the high crystallinity of the structures.

The single PPy nanoribbons with different conductivities and thicknesses synthesized by Chartuprayoon at al. [62], showed successful sensing properties for the detection of ammonia in the concentration range between 0.5 ppm and 50 ppm. The concentration of 0.5 ppm can be considered as the minimum LOD. As the conductivity of the PPy nanostructures is the most important parameter for the sensing performance, the PPy nanoribbons with the conductivity of 0.003 S/cm and 6.5 S/cm showed significant responses to low concentration of ammonia, namely about 8% and 2% for 0.5 ppm and about 15% and 28% for 50 ppm. The response time and recovery time of the sensors were about 8 min and 3 min, respectively, which can be considered very slow times compared to the previously described sensors.

The detection of ammonia was also successful in the case of PPy nanocomposites decorated with various metal oxides (SnO_2_ and ZnO) [13]. In this work the authors found the sensor responses were higher than that observed for the pristine PPy. In addition, a ZnO-based PPy nanocomposite was found to result in a more pronounced response, namely 34%, for 30 ppm of ammonia compared to SnO_2_, which response was only 25% for the same ammonia concentration. This phenomena can be attributed to Zn^2+^ cations that can act as redox-active species and thus increase the charge density and conductivity of the PPy nanocomposite. Considering the response time and recovery time, the faster response time was observed in the case of SnO_2_-based sensor. However, this sensor was not able to recover after three cycles of ammonia exposure, which was opposite to the ZnO-based sensor. The authors explained that reversibility of the ZnO-based sensor is a consequence of its hollow nanotubular morphology, while the SnO_2_/PPy nanostructures had non-hollow morphology, therefore the adsorption and desorption processes happen faster.

One more example of PPy nanostructures decorated with metal oxides is described in the work of Xiang et al. [116]. PPy–graphene nanocomposite decorated with TiO_2_ nanoparticles with diameters of 10–30 nm was used as the sensitive layer of the ammonia sensor. The sensor response to 50 ppm of ammonia was 102.2%, with a response time and recovery time of 36 and 16 s, respectively. Compared with the ZnO-based PPy nanocomposite [13], where the response to 50 ppm of ammonia was about 50%, the PPy/graphene/TiO_2_ nanostructures had much higher sensitivity.

The significant performance of ammonia sensors was recognized in the work of Yan et al. [117]. Compared with the previously described PPy-based sensors, the authors used a special viral-template, based on a genetically modified M13 bacteriophage with a gold-binding peptide. Au nanoparticles were assembled on the template, and these conferred electrical conductivity to the bio- template, what enabled the electropolymerization of the PPy and the formation of Au/PPy nanopeapods. These specific metal–semiconductor, bio-templated nanomaterials had a LOD of 0.007 ppmv, what can be considered as noteworthy sensitivity for nanostructures. The authors claimed that the sensing performances may be influenced by the low thickness of the PPy shell (17.4 nm) along the length of the nanopeapods.

An overview of sensors for the detection of ammonia based on other nanomaterials is provided in the Table 4 and Table 5. If we take into account the sensing performances of the literature reports of sensors shown in these tables, we can see that various gas sensing materials were used for the detection of ammonia, including n-type oxide semiconductors (such as SnO_2_ [119]) and p-type oxide semiconductors (such as CuO [120]). In addition, the mentioned sensing materials can work at high operating temperatures, or even at room temperature. It is difficult to note significant differences in the sensing properties of the described sensors. However, the sensors’ characteristics depends on the type and morphology of the sensing layers. From the group of the metal oxide-based ammonia sensors, we can identify Co_3_O_4_ nanosheet structures as the sensitive layer with the best performance, with the lowest LOD (0.2 ppm) and the most improved response time/recovery times (~9 s/~134 s) [121]. Otherwise, if we consider LOD and response time/recovery times as the major parameters for detection of biomarkers related to different diseases (very low concentrations of target analysis are related to diseases [45,108]), we can conclude that single crystal PPy nanotubes showed the most advanced sensing properties for the detection of ammonia, with LOD (0.00005 ppm) and response/recovery times of less than 16 s/16 s [114]. 

### 4.2. Detection of Other Gases and VOC 

An overview of CP sensors for the detection of VOCs is listed in the Table 6. The chemiresistive sensor based on PPy nanoparticles prepared by Kwon et al. [58] was used for the detection of VOCs such as acetonitrile, acetic acid, and methanol. Significant results were noticed for methanol, where the sensor showed a minimum LOD close to 50 ppm of methanol. The response time of the sensor was less than 1 s. This value can be considered as faster compared with other methanol sensors based on CPs, where the response time was 8 s for 1 ppm of methanol and 2 s for 2000 ppm of methanol [11]. In the case of the recovery time, 90 s was necessary for sensor to recover after exposure to 50 ppm of methanol. On the other hand, the sensor did not show a LOD for acetonitrile and acetic acid as low as in the case of NH_3_ and methanol, namely the minimum LOD was 100 ppm of acetonitrile and acetic acid. The response and recovery times were more or less similar, i.e., less than 1 s and less than 10 s, respectively. Considering the sensing mechanism between the tested VOCs and the PPy sensing layer, it can be presumed that acetonitrile as a weak proton-transfer base (pKa = 25) behaves like ammonia, causing an increase in the electrical resistance, whereas the acetic acid vapor reacts similarly to alcohols, thus causing a decrease in the electrical resistance. The conclusions from this work are that the sensor’s response and the recovery time correlate with the size of the nanoparticles: better response and longer recovery time are observed for sensing layers with smaller nanoparticle diameters. As already explained above, in general the nanostructures with smaller diameter provide a higher surface-to-volume ratio and higher conductivity together with efficient charge carrier transport [129]. The longer recovery time can be explained by a slow desorption of the gas molecules from the PPy nanoparticle backbone.

Besides ammonia, multidimensional PPy nanotubes decorated with nanowires and nanonodules [113] were used for the detection of ethanol. The authors observed a sensor response to ethanol with a minimum LOD of 1 ppm. They found the sensor response for 100 ppm of ethanol was about 4%, which is higher than in the case of a sensor based on pure PANI nanostructures where the sensor response was only around 1% for the same ethanol concentration [8]. However when the PANI nanostructures were decorated with nanosized silver nanoparticles, the sensor response increased up to 18%. The response time of sensors based on multidimensional PPy nanotubes [113] was extremely fast in comparison with PANI based sensors described in [8], i.e., 1 s and 52 s, respectively.

Joulazadeh et al. used nanotubular PPy and nanofibrillar PANI (see Figure 10) for the detection of various alcohols (propanol, butanol, methanol, and ethanol) [53]. Nanostructures of this kind showed a very low LOD to the mentioned alcohols up to 3 ppm. From this work can be concluded that the resistance increase is related to the exposure to the gases. The hydrogen bonding interactions between the polar –OH groups of alcohols and the polymer result in dipole–dipole interactions, and delocalization carriers along the PPy chains which is manifested by the increase of the resistance [87]. PPy exhibited an acceptable performance in the presence of all tested alcohol vapors, however the response and the recovery time varied for different exposed alcohols. The sensor showed higher response to propanol and butanol, what is attributed to the longer alkyl chain comparing to ethanol and methanol, and thus to higher electrophilicity [134]. Methanol and ethanol had shorter recovery times, which is coherent with a faster desorption process due to their smaller molecular size compared with propanol and butanol. This effect of analyte size, namely the length of hydrocarbon chain in aliphatic alcohols, on the sensor response and recovery time was also observed for PANI-based sensors [22]. All types of the abovementioned sensors showed reversible behavior. If we compare both polymers tested in reference [53], the authors concluded that both sensors (PPy and PANI) were able to detect the same minimum limit concentration of alcohols in the range of 3 ppm, but the response of the PANI sensor was higher for all tested alcohols. Also, the PANI sensor had the faster response time to all tested alcohols in comparison with PPy sensor. The better observed response of the PANI sensor can be probably related with the higher surface area-to-volume ratio of the nanostructures, which is manifested in the easy adsorption of the target analytes on the sensitive layer [135]. However, the PPy sensor was able to completely recover and showed a reversible behaviour for all mentioned alcohols, what was not the case of the PANI sensor. This phenomenon can be attributed to the analyte molecules, which are trapped within the interconnected structure of the sensing layer [136].

PPy nanocomposites decorated with SnO_2_ and ZnO oxides [13], besides the detection of ammonia, were also used for the detection of ethanol and methanol. Unfortunately, the tested sensors did not have good response for ethanol and methanol like for ammonia. The sensitivity to 30 ppm of ammonia was about 34% in the case of a PPy/ZnO sensor and 25% in the case of a PPy/SnO_2_ sensor. The best response was shown again the sensor based on PPy decorated with ZnO, namely 3% for 30 ppm of ethanol, and 1.5% for 30 ppm of methanol. The SnO_2_ sensor had responses of 2.4% and 1.3% for the 30 ppm of ethanol and methanol, respectively. The ammonia in the contact with PPy can affect the intrachain conductivity in PPy macromolecules, whereas alcohols, such as methanol and ethanol, can only influence the interchain conductivity process [137]. The authors claimed that the contribution of the intrachain conductivity to the total conductivity of a conducting polymer is much higher than that of the interchain conductivity. Therefore the sensors’ response is expected to be much greater in the presence of ammonia than that of alcohols. 

In the work of Huang and colleagues a sensor was made by a combination of ZnO nanoparticles with mean size of 28 nm and 20 wt% of PANI and tested in the detection of various alcohols and ketones [12]. The sensor showed response times to methanol, ethanol and acetone within 148, 32 and 49 s, respectively, and recovery times within 118, 109 and 160 s, respectively, at an operating temperature of 90 °C. As expected, according to our previous explanation about the influence of the alcohol chain length on the sensitivity, the sensor showed a higher response to ethanol than to methanol. This response to ethanol was also higher than to acetone.

Lee et al. have developed a PPy-based nanostructured sensor combined with gold, which serves as a source for localized surface plasmon resonance (SPR) [10]. Beside the common experimental set-up parts used for chemiresistive gas sensors such as a gas chamber and mass flow controller, their set-up requires in addition a spectrometer and an optical probe which sends the light vertically to the Au–PPy nanorods. The absorbed light was then sent back to the spectrometer through a light detector. The authors tested the detection of three different analytes, namely benzene, toluene and acetic acid. The baseline for the adsorption spectrum was obtained with N_2_. This sensor was able to detect 10 ppm of mentioned VOC, The response and recovery time were 20 and 40 s, respectively.

PPy structures behave as semiconductor materials thus they can be used for the creation of electrical devices which do not need to work by a chemiresistive transduction mechanism. The PPy as p-type semiconducting polymers with a metal contact can form Schottky barriers. Campos et al. presented Schottky barrier devices with heterojunctions between p-doped PPy and n-doped silicon [131]. They used electrochemically polymerized PPy films with thicknesses in the range of 86 to 1140 nm, which were grown on n-silicon working electrodes. The structures were used for the measurements of current density-voltage characteristics (J-V), checking of Schottky barrier diode behavior, capacitance-voltage (C-V) and frequency characteristics of the heterojunction. The measurements were observed under the exposure to air and 10 ppm of acetone. The measurement was based on calculation of current density according to the following equation:

(5) $$J=J_{0}\cdot exp\left(\frac{qV}{nkT}\right),$$

where *J*_0_ is saturation current density, *n* is the ideality factor or quality factor, *V* is the applied voltage, k is the Boltzmann's constant, *q* is the electronic charge, and *T* is the absolute temperature). From the J-V plot, authors observed the changes of the saturation current density from 3.5 × 10^−6^ A/cm^2^ to 2.9 × 10^−6^ A/cm^2^, the ideality factor from 2.3 to 2.1, and the rectification ratio from 4205 to 4370, during the exposure to air and acetone, respectively. The capacitance measurements confirmed the decrease of the saturation current density and an increase in the rectification ratio due to acetone vapor exposure. The PPy/silicon p-n heterojunction behaves as a gas sensor for the detection of 10 ppm of acetone, and the changes of the measured parameters depend on the thickness of the PPy layer.

In other work Campos et al., developed an electrical device for the detection of methanol based on Al/PPy/Au diodes functionalized with dodecylbenzene sulfonic acid (DBSA) [130]. The PPy/DBSA films were prepared via chemical oxidation, where gold and aluminum electrodes were vacuum deposited on the opposite sides of the film. The same as in previous work, the experiments were performed via J-V characteristics and C-V measurements under exposure to nitrogen and methanol, respectively. Therefore, from the changes in the Schottky barrier height and in the carrier concentration of the diodes, which were confirmed by C-V measurements explained that Al/PPy/Au structures are sensitive to 20 ppm of methanol after 10 minutes of exposure. The system needed 6 h to recover.

Impedance measurement at various frequencies could be used for making more selective sensors. Musio et al. designed and constructed PPy-based sensor on patterned electrodes to probe this method [103]. They studied impedance responses to 200 ppm of four different vapours: methanol, acetone, ethyl acetate and ethanol. The sensor was split into resistive and capacitive parts. Both parts were processed into patterns at different frequencies. Impedance was mostly linear, therefore the resistance pattern had no evident frequency dependence but capacitive patterns showed selective detection of ethanol and methanol at low frequencies. 

A better approach on the sensor response was demonstrated by Bhatt and Jampana by measuring the changes in its capacitance, resistance and the dissipation factor upon exposure to organic volatiles such as acetone, ethanol and isopropyl alcohol [102]. An interdigitated structure with gold electrodes 20 µm wide and 10 µm spaced with electrochemically deposited PPy was tested at range of 10 Hz to 2 MHz. They showed that the dissipation factor (DF), which is a ratio of the resistive and absolute value of the reactive component of impedance, presents peaks at several frequencies corresponding to the resonance frequencies of molecules binding to the PPy. The measurement was provided at high concentration from 10.000 ppm but presented a processing method that is promising to discriminate between chemicals. The magnitude of the dissipation factor was found to be linearly dependent on the VOC concentration. An overview of the sensors based on the other nanomaterials used for detection of VOC and gases is listed in the Table 7. According to the literature overview reported in this table, we can notice that metal oxide-based sensors are able to detect lower concentrations of ethanol and methanol then PPy-based sensors. However, the metal oxide sensors require the quite high operating temperature (180–350 °C). In the case of ethanol, multidimensional PPy nanotubes have the lowest LOD (1 ppm) [113] among the CP group, but considering the metal oxide sensors, MoO_3_/WO_3_ composite nanostructures gave a response to 0.1 ppm of ethanol [139]. On the other hand, the best response time/recovery time is noticed for multidimensional PPy nanotubes (<1 s/4–5 s) [113], so we can conclude this sensor is the fastest one among the sensors described in this work. In the case of methanol, the best results are observed for the sensor based on porous In_2_O_3_ nanobelts, where the LOD was 0.1 ppm and response time/recovery time were approximately about 10 s/10 s for 20 ppm of methanol [101].

### 4.3. Detection of Humidity

Beside the detection of the gases and VOCs mentioned above, researchers have found applications of PPy-based sensors for humidity detection [143]. Indeed, the sensitivity to humidity (water vapour) is a very important parameter because it is the most significant interfering vapour. Some works focused on the relative humidity sensing are briefly presented in the following paragraphs. Joulazadeh et al. studied the humidity sensing performances for the previously described PPy nanostructures synthesized via anhydrous FeCl_3_ and methyl orange [64]. The tubular morphology of PPy nanostructures showed positive changes of the resistance (R > R_0_) for a low humidity concentration (25 ppm of water vapour), and negative changes (R < R_0_) for higher concentrations (56 ppm of water vapour). The authors explained that the increasing and decreasing resistance for different concentrations of humidity for PPy is affected by proton exchanges between the water molecules and the polymer NH_2_+ groups and by the swelling effect of the polymer chains. The same behaviour is expected for PANI-based sensors [144,145]. The increase in resistance at lower concentrations can be explained by the greater distances in interchain connectivity of the polymer network [9]. The proton transfer between the water molecules and NH_2_^+^ is not feasible due to the swelling phenomenon of polymer chains which increases the distance between the chains. In the case of the higher humidity concentration, the resistance increases at the beginning which is related with effect above described, but at one point it started to decrease, as a consequence of water molecules absorbed on the surface of the polymer, enabling the transfer of protons. 

Lin et al. used impedance sensors for the measurement of humidity [146]. The analysis was based on measurement of resistance and capacitance by an AC voltage with an amplitude of 1 V. The response time or recovery time are defined as the times required for the impedance of a sensor to change by 90% of the total impedance. The sensitive layer of the sensor was based on different amounts of graphene composited with PPy structures, where polymerization of the PPy layer is done via chemical oxidation. The authors checked the impedance values versus relative humidity (RH), where the samples were exposed to RH in the range from 12% to 90%. The humidity sensitivity is defined as:

(6) $$S=\frac{R_{d}}{R_{h}},$$

where *R*_d_ and *R*_h_ were the impedance at 12% RH and at 90% RH, respectively. PPy sensitivity to RH is poor with an impedance in range 2–2.5 × 10^7^ Ω, where the sensitivity is 60 at 90% RH. The sensor made from 10% graphene/PPy showed the greatest sensitivity to humidity (S = 138 at 90% RH). On the other hand, this structure had an efficient response time and recovery time, 15 s as the RH was increased 12%–70%, and 20 s as RH decreased from 70% to 12%, respectively. This response time and recovery time were shorter in comparison with those reported in the works of Joulazadeh et al. [143], where the response time was 41 s when the RH changed from 11% to 95%, and the recovery time was 120 for RH changes from 95% to 11% RH. The authors suggested the potential application of graphene/PPy composite in the fabrication of high-performance humidity sensors. 

The work of Yang et al. presented a MEMS helix interdigitated electrode chemically coated by PPy with grain diameters of about 0.3–0.5 μm making the film porous [147]. CMOS electronics integrated within the MEMS structure form a ring oscillator as the sensing circuits. Capacitance changes are transduced to frequency. The work presents a logarithmic dependency of frequency on RH in the range 32 MHz/80%RH to 38.5 MHz/25%RH and sensitivity 99 kHz/%RH at 25 °C. Temperature dependency was also demonstrated, decreasing to 32 kHz/%RH at 75 °C.

## 5. Conclusions

In this review, we have described the synthesis of PPy-based ammonia and VOC sensors in detail and compared their sensing effectivity with other CP, primarily with PANI, as well as with other well-known gas sensing materials such as metal oxides. We showed all necessary synthesis parameters together with the type of reactants such as a polymer precursor and an oxidant agent for a successful preparation of polymer sensing layer. We explained the uniformity and homogeneity of polymer nanostructures depend strongly on the type of synthesis, eventually application of template, which ensures the ordering of polymer nanostructures. According to our literature survey, the majority of research works were devoted to the electrochemical synthesis approach combined with various templates. 

The prepared polymer nanostructures can be of different shapes such as nanoparticles, nanorods, nanowires, nanotubes, nanoplates, nanoribbons, etc. Concerning the nanostructure size, one can conclude the smaller size can provide increased surface to volume ratio, i.e., higher active surface area and thus higher sensitivity. However, the recovery time of a sensor with smaller size of nanostructures was found to be longer than in the case of bigger polymer structures. Beside polymer size and morphology, its functionalization with various nanosized materials like noble metals, metal oxides and different types of carbon was found to have a crucial effect on the final sensitivity and usually resulted in enhanced sensor performance. 

It is obvious that the overall polymer morphology has also a significant influence on the sensitivity performance as we showed in few summarizing tables: the lowest LOD in the ppb level was found for single crystal PPy nanotube sensors for ammonia. This LOD meets the requirement for practical sensor applications in the analysis of human breath and disease diagnostics. Such a low detectable concentration of ammonia was not achieved with metal oxide-based gas sensors, where the best LOD was found to be 0.2 ppm for a Co_3_O_4_ nanostructured sensor working at room temperature, and V_2_O_5_ sensors operating at temperature of 350 °C. Beside the sensitivity as a key parameter representing the sensor efficiency, one has also to take into account the sensor response and recovery time. We can conclude that performance of PPy-based sensors in the case of ammonia detection is really enticing since their fastest response and recovery times are found to be less than 1 s and 2 s, respectively, for 5 ppm of ammonia. In the case of alcohol sensing, the lowest LOD was found to be 1 ppm for ethanol using a chemiresistive sensor with PPy nanotubes and 1 ppm for methanol using a PPy-coated quartz fiber optical sensor. These limits of detection were 10× surpassed with metal oxide based sensors, where the lowest concentration of 0.1 ppm was detected for methanol and ethanol using sensing layers fabricated from composites of SnO_2_, WO_3_ and In_2_O_3._

The review showed that gas and VOC sensors based on PPy working on a chemiresistive transducing principle have received much more attention than the other ones. PPy sensors were most frequently tested for the detection of ammonia and several type of alcohols. In general, the PPy sensor response was higher to ammonia than to other gases and VOC. This is due to the electron-donating behavior of ammonia and its high affinity to the PPy structure. This observation indicates the future perspectives in the application of this sensor and can further expand the research focus towards detection of other electron-donating molecules which have not been sufficiently studied so far. 

Even though CP-based sensors can be tailored for particular properties, easily processed and selected to be inert in the environment containing the analyte, still there are some remaining challenges which have to be addressed. The main drawback of these sensors lies in their irreversibility which is believed to be caused by nucleophilic attack of the analytes on the carbon backbone. The other disadvantage is a short sensor lifetime. The sensing properties of CP-based sensors as well as the other sensors are significantly influenced by the ambient conditions, such as temperature and humidity. In addition, a swelling effect usually occurs in CP layers, which can cause electrical resistance changes in chemiresistive sensors. Therefore, in order to successfully make use of CP-based gas and VOC sensors in medical applications, the development of the sensor market has to aim at emerging technologies employing smart and cheap flexible sensors with long-term stability of the sensing material.

## Figures and Tables

**Table 1:** FT-IR spectroscopic analysis of PPy.

Vibration of the PPy Structure	Wavenumber (cm^−1^) of PPy Prominent Peaks
C–H stretching	2854–2931 [54]
C–H in plane deformation vibration	1039–1220 [54,55]
C–H out-of-plane vibration	804–931 [55,56]
C–H wagging vibration	782 [54]
C=C stretching of pyrrole ring	1538–1553 [17,57]
C–N	1192 [57] 1484 [54]
N–H stretching	3432–3443 [17]

**Table 2:** Overview of preparation methods of PPy nanostructured sensing layer.

Nanostructures Morphology	Type of Substrate	Fabrication Process	Oxidation Agent	Ref.
Nanowires diameter: 50 nm	Silicon	AAO template assisted electrochemical polymerisation in potentiostatic mode at 1 V	Lithium perchlorate LiClO_4_	[21]
Nanobelts, nanosheets and nanobricks with diameter of 400 nm	Stainless steel foil	Electrochemical polymerisation in potentiodynamic mode cycling from 0 to +1.2 V	Potassium nitrate KNO_3_	[61]
Nanoribbons length of 1 cm and diameter of	Silicon	Ni nanobands assisted electrochemical polymerisation in potentiostatic mode at 0.7 V	Lithium perchlorate LiClO_4_	[62]
Nanorods of Au/PPy diameter: 200 nm	Glass	AAO template assisted electrochemical polymerisation in potentiostatic mode at 0.95 V	Tetraethyl-ammonium tetrafluoroborate (C_2_H_5_)_4_N(BF_4_)	[10]
Nanotube diameter: 50 nm	Glass	Soft template assisted chemical polymerization	Ferric chloride FeCl_3_	[53]
Nanowires diameter: 300 nm	Silicon with SiO_2_ layer	AAO template assisted chemical polymerisation	Ferric chloride FeCl_3_	[60]
Nanoparticles diameter: 20, 60, 100 nm	Glass	Chemical polymerization	Ferric chloride FeCl_3_	[58,59]
Globular structures with diameter of about 590 nm	Printed circuit board	Chemical polymerization	Ammonium peroxydisulfate (NH_4_)_2_S_2_O_8_ or ferric chloride FeCl_3_	[68,69]
Nanolayers with thickness of 37, 43, 62, and 71 nm	Various polymeric substrates	Vapour-phase polymerization	Ferric chloride FeCl_3_	[70]
Compact layers (thickness N/A)	Cellulosic paper	“Pen-writing” vapour-phase polymerization	Ferric chloride FeCl_3_	[71]

**Table 3:** Overview of PPy used as active layers for detection of ammonia at room temperature.

Polymer Type	LOD	Response Time/Recovery Time	Transducing Mechanism	Ref.
PPy nanoparticles	5 ppm	Less than 1 s/2 s	Chemiresistive	[58]
Multidimensional PPy nanotubes	0.01 ppm	Less than 1 s/55–60 s	Chemiresistive	[113]
Single PPy nanowire	40 ppm	15–10 min (for 40–300 ppm)/15 min for 40 ppm	Chemiresistive	[36]
PPy nanowires	1.5 ppm	60 s for 73 ppm/prolonged with increasing of con. (1.5–73 ppm)	Chemiresistive	[18]
Single crystal PPy nanotube	0.00005 ppm	~16 s/~16 s for 1 ppm	Chemiresistive	[114]
PPy nanoribbons	0.5 ppm	~8 min/3 min	Chemiresistive	[62]
PPy nanotubes PPy/Ag–AgCl composite Nanotubes	–	>1000 s for 100 ppm/−150 s for 100 ppm/500 s	Chemiresistive	[115]
PPy/ZnO nanocomposite PPy/SnO_2_ nanocomposite	10 ppm	~100 s for 24 ppm/100 s ~50 s for 24 ppm/250 s for first 3 cycles	Chemiresistive	[13]
PPy/graphene nanocomposite decorated with TiO_2_ nanoparticles	1 ppm	~36 s/~16 s for 50 ppm	Chemiresistive	[116]
Au/PPy nanopeapods	0.007 ppm_v_	~15 min for 5 ppm_v_/did not reach R_o_ value	Chemiresistive	[117]

**Table 4:** Overview of different nanomaterials used as active layers for detection of ammonia at room temperature.

Polymer Type	LOD	Response Time/Recovery Time	Transducing Mechanism	Ref.
Nanofibrous PANI films	5 ppm	~200 s/~100 s	Chemiresistive	[122]
PbS quantum dots/TiO_2_ nanotube	2 ppm	-/-	Chemiresistive	[121]
Co_3_O_4_ nanosheets	0.2 ppm	~9 s/~134 s	Chemiresistive	[121]
Carbon nanotubes/SnO_2_ nanocomposite	10 ppm	~100 s/~192 s	Chemiresistive	[123]
CuO Nanostructures	50 ppm	~6 min/~5–6 min	Chemiresistive	[120]
Au-decorated tungsten oxide nanoneedles	-	~4 s/~4 min for 100 ppm	Optical	[124]

**Table 5:** Overview of different nanomaterials used as active layers for detection of ammonia at higher temperature.

Type of Material	LOD	Response Time/Recovery Time	Operative Temperature	Transducing mechanism	Ref.
V_2_O_5_ and V_7_O_16_ thin-film structures	0.2 ppm	~1 h/~2 h	350 °C	Chemiresistive	[125]
SnO_2_-Nb-Pt nanocrystaline	10 ppm	~150 s/~170 s	355 °C	Chemiresistive	[126]
Nanoporous NiO thin films	20 ppm	~89 s/~128 s	250 °C	Chemiresistive	[127]
Pt activated SnO_2_ nanoparticle clusters	10 ppm	~75 s/~67 s for 50 ppm	115 °C	Chemiresistive	[119]
Mixed WO_3_–SnO_2_ nanostructures	0.52 ppm	~220 s/~195 s for 400 ppm	200 °C	Chemiresistive	[128]

**Table 6:** Overview of CPs used as active layers for detection of other gases and VOCs.

Polymer Type	Target Analytes	LOD	Response Time/Recovery Time	Transducing Mechanism	Ref.
PPy nanoparticles	Methanol Acetonitrile Acetic acid	50 ppm 100 ppm 100 ppm	1 s/90 s <1 s/<10 s <1 s/<10 s	Chemiresistive	[58]
PANI/Pd Nanocomposite	Methanol	1 ppm	~8 s/~9 s	Chemiresistive	[11]
Multidimensional PPy nanotubes	Ethanol	1 ppm	< 1 s/4–5 s	Chemiresistive	[113]
Nanotubular PPy	Butanol Propanol Methanol Ethanol	3 ppm for all alcohols	Data for 10 ppm: ~200 s/>5 s ~200 s/>5 s ~150 s/5 s ~110 s/5 s	Chemiresistive	[53]
Nanofibrillar PANI	Butanol Propanol Methanol Ethanol	3 ppm for all alcohols	Data for 10 ppm: ~100 s/not completely recovered ~80 s/not completely recovered ~80 s/~15 s ~80 s/~15 s	Chemiresistive	[53]
Au/PPy nanorods	Benzene Toluene Acetic acid	10 ppm for all analytes	20 s/40 s	Optical based on localized surface plasmon resonance	[10]
PPy coated quartz fibres	Methanol Ethanol Acetone Toluene Chloroform Isopropyl alcohol	1 ppm for methanol 10–30 ppm for other VOC	Data for 286 ppm of methanol: 200 s/400 s. Data for 6 ppm of methanol: 100 s/200 s.	Optical based on reflectance	[130]
Al/PPy/Au/ dodecylbenzene sulfonic acid diodes	Methanol	20 ppm	10 min/6 h	Capacitive	[131]
PPy films on n-silicon	Acetone	10 ppm	-/-	Capacitive	[132]
Single PPy nanowire	Heptanal Acetophenone Isopropyl myristate 2-Propanol	8.982 ppm 798 ppb 134 ppm 129.5 ppm	-/-	Chemiresistive	[133]
PPy film on gold IDE/FR4	Acetone Ethanol Isopropyl alcohol	-	-/-	Impedance	[102]
PPy film on gold	Methanol Acetone Ethyl acetate Ethanol	-	~100 s/~50 s	Impedance	[103]

**Table 7:** Overview of different nanomaterials used as active layers for detection of other gases and VOC.

Type of Material	Target Analytes	LOD	Response Time/Recovery Time	Operative Temperature	Transducing Mechanism	Ref.
Mixed WO_3_–SnO_2_ nanostructures	Ethanol	0.131 ppm	~225 s/~300 s for 180 ppm	300 °C	Chemiresistive	[128]
Crystalline/amorphous core/shell MoO_3_ nanocomposite	Ethanol	10 ppm	<40 s/<40 s	180 °C	Chemiresistive	[138]
MoO_3_/WO_3_ composite nanostructures	Ethanol	0.5 ppm	~13 s/~10 s	320 °C	Chemiresistive	[139]
SnO_2_-Pd-Pt-In_2_O_3_ composite	Methanol	0.1 ppm	~32 s/~47 s for 100 ppm	160 °C	Chemiresistive	[140]
Porous In_2_O_3_ nanobelts	Methanol	0.1 ppm	~10 s/~10 s for 20 ppm	370 °C	Chemiresistive	[141]
SnO_2_-ZnO composite nanofibers	Methanol	1 ppm tested	~20 s/~40 s for 10 ppm	350 °C	Chemiresistive	[142]
